# Supplementary material for: Intrinsic and extrinsic cues time somite progenitor contribution to the vertebrate primary body axis
Source: eLife. 2024 Jan 9;13:e90499. doi: 10.7554/eLife.90499 (PMC10834026; doi:10.7554/eLife.90499)
Supplement: Supplementary file 1. — (a) Differential gene expression (clusters 0–13). Differential expression analysis was performed in Seurat to identify ‘markers’ of each cluster, that is, those genes whose expression differed significantly between a given cluster and the remainder of the dataset. For each cluster, the 10s genes with the most significant (smallest) adjusted p-value are provided in this table. (b) GO term analysis for cluster 6. GeneCodis was used to perform Gene Ontology analysis on the full list of differentially expressed genes in cluster 6 conserved across samples (i.e. genes differentially expressed in cluster 6 across all three samples). (c) Differential gene expression for HH8- and HH4-derived cells in clusters 6, 8, and 12. Pooled HH8- and HH4-derived cells in the central clusters of the UMAP plot were used to test for genes differentially expressed between these groups. Cell cycle-related genes were removed from the analysis. (d) Primer sequences for RT-qPCR. [file elife-90499-supp1.docx]

*Supplementary File 1a*

| Cluster | Gene | Log_2_(Fold-Change) | Adjusted p-value |
| --- | --- | --- | --- |
| 0 | *HOXA7* | 1.9496338890655 | 0 |
| 0 | *eGFP* | 1.79926919223305 | 0 |
| 0 | *HOXB8* | 1.70319522234973 | 0 |
| 0 | *CDX4* | 1.59300323388873 | 0 |
| 0 | *MSX1* | 1.36679666722387 | 2.46628686800213E-295 |
| 0 | *ATP2B1* | 0.9505805990838 | 1.42802105117689E-257 |
| 0 | *WNT5A* | 1.05362309733373 | 1.04864804124596E-243 |
| 0 | *FGF19* | 1.23946510195106 | 1.37033716161311E-237 |
| 0 | *ID1* | 1.12987314753611 | 5.25102458527783E-226 |
| 0 | *Pou5f3* | -1.38972912656039 | 2.36221422404344E-218 |
| 1 | *MIXL1* | 3.58795746206821 | 0 |
| 1 | *CER1* | 2.87849110404204 | 0 |
| 1 | *GSC* | 2.51944790052532 | 0 |
| 1 | *CHRD* | 2.20958320983799 | 0 |
| 1 | *LDHA* | 2.13917827613479 | 0 |
| 1 | *EOMES* | 2.11133471305459 | 0 |
| 1 | *HHEX* | 1.70798541915079 | 0 |
| 1 | *CFC1* | 1.6906566684956 | 0 |
| 1 | *STK26* | 1.65314571843814 | 0 |
| 1 | *GATA6* | 1.57995715798053 | 0 |
| 2 | *AGRP* | 2.05116123467884 | 0 |
| 2 | *MSGN1* | 2.01742248398195 | 0 |
| 2 | *CXCL14* | 1.50158277610382 | 1.32053929591911E-276 |
| 2 | *DLL1* | 1.16586506142267 | 1.7443439310959E-253 |
| 2 | *ALDH1A2* | 1.41866777284285 | 6.04971852125113E-242 |
| 2 | *MSX1* | -2.52904115471438 | 6.37662024762309E-237 |
| 2 | *KRAS* | 0.82770791949726 | 9.96915154395077E-217 |
| 2 | *RNH1* | 1.08605058601933 | 8.65638198126585E-206 |
| 2 | *RDH10* | 1.04307059206148 | 6.36102174754014E-191 |
| 2 | *SGPL1* | 0.817201414934171 | 2.82128783860915E-190 |
| 3 | *GBX2* | 1.53817472516901 | 0 |
| 3 | *GJA1* | 1.38835536010913 | 0 |
| 3 | *EDNRB* | 1.20009455194226 | 2.52478672682819E-289 |
| 3 | *CLDN1* | 1.20413809594401 | 1.17935128903501E-277 |
| 3 | *CDH11* | 1.25112401401076 | 6.25834013605549E-257 |
| 3 | *SOX3* | 1.54211085212976 | 3.17635246464633E-247 |
| 3 | *HINTW* | 1.92943556803832 | 3.02369221987231E-199 |
| 3 | *PDGFA* | 0.986728347014766 | 5.40924194099481E-195 |
| 3 | *CTTNBP2* | 0.866668411323754 | 4.78619642503906E-191 |
| 3 | *AP1S3* | 0.806097731269968 | 1.35496510194021E-175 |
| 4 | *WNT5A* | 1.19230310822398 | 1.46284545077589E-183 |
| 4 | *eGFP* | 1.47190878778027 | 5.42969070297272E-178 |
| 4 | *CDX4* | 1.28396931508265 | 4.7994468946369E-171 |
| 4 | *HOXB8* | 1.38798865712259 | 1.33901112440224E-170 |
| 4 | *MSX1* | 1.23406164719858 | 1.66593707144748E-166 |
| 4 | *FGF19* | 1.31913947173534 | 5.1881920449563E-165 |
| 4 | *KPNA2* | 1.1244865752887 | 1.08515343694279E-164 |
| 4 | *HOXA7* | 1.44451732868615 | 7.38455521897304E-156 |
| 4 | *TMSB4X* | -1.18765913768026 | 1.25741984738072E-148 |
| 4 | *TCF12* | 0.689377680633102 | 3.7014579463651E-145 |
| 5 | *MSGN1* | 2.06036738777217 | 2.75041310311928E-206 |
| 5 | *ALDH1A2* | 1.8077664815224 | 4.16103222583803E-205 |
| 5 | *AGRP* | 1.80310854909397 | 4.34016209682778E-174 |
| 5 | *RDH10* | 1.30971797527668 | 5.48815068084915E-165 |
| 5 | *CENPF* | 1.36649903527463 | 8.29532481213387E-162 |
| 5 | *DLL1* | 1.00958582280298 | 5.1844140060902E-137 |
| 5 | *RGS3* | 0.780991833343416 | 9.84168966965528E-130 |
| 5 | *RNH1* | 1.10060407980127 | 1.93676703344941E-128 |
| 5 | *MSX1* | -2.42204659813208 | 2.73078300315636E-127 |
| 5 | *HMGB2* | 0.56896092368797 | 1.92833812621915E-122 |
| 6 | *TNNT2* | 0.417121158385769 | 3.99299369410458E-190 |
| 6 | *PMP22* | 0.263415775764942 | 1.36122866810487E-146 |
| 6 | *RN7SL1* | 0.846911374647191 | 7.36609061716575E-144 |
| 6 | *RAMP3* | 0.290333547890103 | 3.09950133586924E-143 |
| 6 | *ARMC7* | 0.341010996351886 | 6.62616408602338E-143 |
| 6 | *RGS3* | -0.294855602676253 | 9.8465042510006E-133 |
| 6 | *ASCC3* | -0.300076531691926 | 2.1927912379702E-131 |
| 6 | *LHX1* | 0.298536165933243 | 8.26926129288258E-130 |
| 6 | *HS6ST2* | -0.307576179937828 | 3.04263269316031E-129 |
| 6 | *ZCCHC6* | -0.349285088332166 | 5.78347157870883E-129 |
| 7 | *FABP7* | 2.31681516022095 | 1.60283532836868E-187 |
| 7 | *DLX5* | 2.20899844325514 | 2.92801657280801E-183 |
| 7 | *MSX2* | 2.16202382247032 | 1.70293073811211E-181 |
| 7 | *TMSB4X* | -1.61409782658695 | 1.35357226080171E-164 |
| 7 | *LDHB* | 1.10121559919432 | 1.15773661022506E-148 |
| 7 | *HOXA7* | 1.97308338168153 | 7.02283658518382E-143 |
| 7 | *TFAP2A* | 1.9970154846481 | 4.28029629795172E-133 |
| 7 | *CLDN3* | 1.70572427249482 | 5.99432961917036E-129 |
| 7 | *COL1A2* | -2.39408795305034 | 2.11770458415652E-127 |
| 7 | *CDH2* | -2.17845396127837 | 2.69935264062083E-127 |
| 8 | *PPIA* | -0.646872763502564 | 6.35709080058737E-57 |
| 8 | *MRPL34* | -0.645923944211213 | 7.42362574098425E-56 |
| 8 | *MRPL41* | -0.553041183770016 | 3.52860856240581E-51 |
| 8 | *FABP5* | -0.370293164107938 | 3.25608419183137E-44 |
| 8 | *RAC1* | -0.47358803496502 | 2.0412957765153E-43 |
| 8 | *DGCR6* | -0.509463531405073 | 1.38099324730152E-40 |
| 8 | *MED6* | -0.375431679312548 | 3.16267361411543E-40 |
| 8 | *PSMA1* | -0.678703160750433 | 1.50007582355636E-39 |
| 8 | *DCTN2* | -0.415042168137644 | 3.43297847916506E-39 |
| 8 | *IMP4* | -0.350031992402085 | 1.22684411592149E-38 |
| 9 | *GSC* | 0.250722057211903 | 1.13221965643841E-41 |
| 9 | *CHRD* | 0.941430119116635 | 9.30065811856524E-38 |
| 9 | *EOMES* | 0.450490399351236 | 5.94507832012396E-30 |
| 9 | *STK26* | 0.476404665076204 | 5.49113305755186E-26 |
| 9 | *LDHA* | 0.391888595241813 | 2.04436593524018E-25 |
| 9 | *SOX17* | -0.955514622045934 | 1.36362331166111E-24 |
| 9 | *EXFABP* | 0.290329737818581 | 4.48248603254064E-23 |
| 9 | *SMS* | 0.614485826748932 | 2.06556013454308E-22 |
| 9 | *SFRP1* | 0.533561232121688 | 1.18075121793058E-19 |
| 9 | *IGFBP2* | 0.473427770760102 | 7.88385397672752E-19 |
| 10 | *SGK1* | 1.45640915817001 | 7.17863561437477E-92 |
| 10 | *ASTL* | 1.7810786026116 | 2.36792407275542E-91 |
| 10 | *CA2* | 1.67889652222983 | 1.23377942158736E-90 |
| 10 | *ERNI* | 1.0261144180689 | 1.77186631446483E-78 |
| 10 | *ANO3* | 1.24597707381906 | 3.44671185736367E-75 |
| 10 | *IGFBP2* | 1.21811855815857 | 6.51868675555751E-73 |
| 10 | *GNOT2* | 1.53647955217464 | 1.8757262825897E-72 |
| 10 | *ENS-3* | 1.07858965484008 | 8.21057589100296E-72 |
| 10 | *LDHA* | 1.57489934743726 | 2.89385437448538E-71 |
| 10 | *CDH2* | -2.44921443649737 | 5.85103897417772E-67 |
| 11 | *AvBD10* | 0.657018233264079 | 2.18047858731018E-213 |
| 11 | *LOC395991* | 4.4703934824971 | 3.54211066386537E-109 |
| 11 | *SOX17* | 4.30996078488594 | 1.81787512224253E-107 |
| 11 | *KRT7* | 3.13721369585305 | 7.06395698223179E-105 |
| 11 | *APOA1* | 4.62766475345107 | 1.78799713527858E-101 |
| 11 | *GATA5* | 1.86992678649693 | 1.35096599084095E-90 |
| 11 | *CXCR4* | 2.95181586119202 | 6.16718179324134E-90 |
| 11 | *TMSB4X* | 1.60408285895032 | 1.05032205194193E-88 |
| 11 | *CLDN3* | 2.2447134706466 | 6.57242344304436E-82 |
| 11 | *EZR* | 1.85143102060713 | 2.01483255728218E-78 |
| 12 | *MYH10* | -1.35919412913327 | 9.39564883180538E-39 |
| 12 | *LFNG* | -1.7372403956411 | 3.16256211639243E-38 |
| 12 | *PRTG* | -1.64626561038074 | 2.07081618464162E-36 |
| 12 | *CDH2* | -1.95491333679793 | 1.94056836881241E-35 |
| 12 | *ACVR2B* | -1.43355881903305 | 3.39718547938866E-35 |
| 12 | *GJA1* | -1.7623543058503 | 1.62977613299892E-34 |
| 12 | *COL1A2* | -1.93004311676896 | 1.0980575507006E-32 |
| 12 | *LBR* | -1.35089972372763 | 1.8627615931053E-32 |
| 12 | *HYOU1* | -1.20072948867266 | 1.87925377459531E-31 |
| 12 | *HSP90B1* | -1.50779986664999 | 2.93615244002362E-31 |
| 13 | *AGRP* | 0.71802128124676 | 9.22438153673927E-06 |
| 13 | *MSGN1* | 0.437497543184769 | 1.62259748570164E-05 |
| 13 | *MEOX1* | 0.294754624518752 | 0.000100170763316343 |
| 13 | *ZEB2* | 0.362640785204003 | 0.000278741824675477 |
| 13 | *SETD6* | 0.413337203221678 | 0.000615871467978752 |
| 13 | *eGFP* | 0.504494533251947 | 0.00209586528952035 |
| 13 | *TCF15* | 0.345905016395818 | 0.00659844205768626 |
| 13 | *ALDH1A2* | 0.256322744864254 | 0.00817806379401717 |
| 13 | *TSPAN18* | 0.362135032863625 | 0.0197754182627344 |
| 13 | *SMCO4* | 0.318228624638086 | 0.0215366480892156 |

*Supplementary File 1b*

| **GO Term** | **Annotation ID** | **Genes found** | **Input size** | **Term genes** | **Adjusted p-value** | **Relative enrichment** | **genes** |
| --- | --- | --- | --- | --- | --- | --- | --- |
| *cell adhesion* | *GO:0007155* | *21* | *233* | *181* | *0.000368* | *3.593697* | *CDH11, ADAM9, PODXL, EMB, CLDN3, PTK7, PRTG, BSG, SPON1, NCAM1, NRXN1, NFASC, EPHA4, ITGB1, FN1, CDH2, RHOB, PCDH19, APP, AXL, ZYX* |
| *somitogenesis* | *GO:0001756* | *7* | *233* | *27* | *0.010297* | *8.030361* | *POFUT1, ROR2, PTN, LFNG, EPHA4, WNT3A, MSGN1* |
| *positive regulation of astrocyte differentiation* | *GO:0048711* | *3* | *233* | *4* | *0.044944* | *23.230687* | *BIN1, NOTCH1, SERPINE2* |
| *axon guidance* | *GO:0007411* | *10* | *233* | *76* | *0.044944* | *4.075559* | *B3GNT2, EMB, PRTG, BSG, OTX2, NFASC, EPHA4, NOTCH1, EPHA5, WNT3A* |
| *regulation of Notch signaling pathway* | *GO:0008593* | *4* | *233* | *10* | *0.046009* | *12.389699* | *POFUT1, LFNG, ADAM10, NOTCH1* |
| *regulation of axonogenesis* | *GO:0050770* | *4* | *233* | *11* | *0.058729* | *11.263363* | *SIPA1L1, EPHA4, CDH2, WNT3A* |
| *homophilic cell adhesion via plasma membrane adhesion molecules* | *GO:0007156* | *7* | *233* | *50* | *0.089415* | *4.336395* | *CDH11, PRTG, BSG, NCAM1, NFASC, CDH2, PCDH19* |
| *neuroligin clustering involved in postsynaptic membrane assembly* | *GO:0097118* | *2* | *233* | *2* | *0.089415* | *30.974249* | *NRXN1, CDH2* |
| *trigeminal ganglion structural organization* | *GO:0061563* | *2* | *233* | *2* | *0.089415* | *30.974249* | *SLIT1, CDH2* |
| *axis elongation involved in somitogenesis* | *GO:0090245* | *2* | *233* | *2* | *0.089415* | *30.974249* | *FN1, WNT3A* |

*Supplementary File 1c*

| **Gene** | **Log_2_FC HH4** | **Log_2_FC HH8** | **Adjusted p-value** |
| --- | --- | --- | --- |
| *HOXA7* | -4.56075396166316 | 4.56075396166316 | 7.74022587793996E-91 |
| *HOXB8* | -3.63126237573027 | 3.63126237573027 | 9.49944840703166E-91 |
| *MSX1* | -2.76035512958332 | 2.76035512958332 | 4.08265956222547E-78 |
| *CDX4* | -2.57421401161646 | 2.57421401161646 | 2.22292436776557E-75 |
| *FABP7* | -2.76363763027707 | 2.76363763027707 | 6.3404508832246E-69 |
| *FOXD3* | 1.51917404659086 | -1.51917404659086 | 7.04713825386027E-68 |
| *LDHB* | -1.27359191292784 | 1.27359191292784 | 2.63780145221445E-65 |
| *ID1* | -2.19062193003862 | 2.19062193003862 | 2.12348335957229E-64 |
| *WNT5A* | -1.75776508823501 | 1.75776508823501 | 1.55421818551336E-58 |
| *CHRD* | 2.70498887054941 | -2.70498887054941 | 6.50943821365007E-56 |
| *OAT* | -1.34951897680759 | 1.34951897680759 | 3.94106900443992E-51 |
| *LIN28A* | -1.37655334224822 | 1.37655334224822 | 8.14061416764603E-51 |
| *APCDD1* | -0.961433388217504 | 0.961433388217504 | 7.04547923207708E-49 |
| *ATP2B1* | -1.37968001741039 | 1.37968001741039 | 1.01763116962531E-47 |
| *MSX2* | -1.69993275312845 | 1.69993275312845 | 2.74151315923146E-46 |
| *HOXA4* | -1.33029719732012 | 1.33029719732012 | 3.3965748787443E-46 |
| *PRTG* | -1.319886716506 | 1.319886716506 | 2.5267805529959E-45 |
| *HOXA6* | -1.22752725777529 | 1.22752725777529 | 9.3010822596714E-42 |
| *eGFP* | -1.23150144669165 | 1.23150144669165 | 9.35961690536248E-42 |
| *YBX1* | -0.578258359335501 | 0.578258359335501 | 1.34213790988968E-41 |
| *FGF19* | -1.4207965986741 | 1.4207965986741 | 1.9127270213518E-40 |
| *EOMES* | 1.68506260682122 | -1.68506260682122 | 2.42215934533388E-39 |
| *AGRP* | 2.51538286230997 | -2.51538286230997 | 7.71380227137366E-39 |
| *TFDP2* | -1.10531603697908 | 1.10531603697908 | 3.88310406075555E-38 |
| *HOXA5* | -1.19605001732345 | 1.19605001732345 | 1.62788261219325E-37 |

*Supplementary File 1d*

| **Gene** | **Forward primer (5’-3’)** | **Reverse primer (5’-3’)** |
| --- | --- | --- |
| *ACTB* | CAGGTCATCACCATTGGCAAT | GCATACAGATCCTTACGGATATCCA |
| *Hoxa1* | CCTACAACCATGCCCTGTCT | CTTGGTGGTGAAGTTGGTCC |
| *Hoxa2* | AGGCAAGTGAAGGTCTGGTT | GGTCCTCCGAGCCCTTAAAT |
| *Hoxa3* | GCGCTTCCTGAATTCTTCCC | GCGCTTCCTGAATTCTTCCC |
| *Hoxa6* | CTTACACCCGCTACCAAACG | CGGGCTGGGTGGAATTTATG |
| *Hoxb1* | TCGATAGCCTCACGTCTTGG | TCTGGTGGTAAAGGGTAGCA |
